# Esophageal hirudiniasis: an unusual cause of upper gastrointestinal bleeding

**DOI:** 10.2144/fsoa-2022-0023

**Published:** 2022-06-27

**Authors:** Moufida Mahmoudi, Sahar Nasr, Amal Khsiba, Mouna Medhioub, Lamine Hamzaoui, Mohamed Mssadek Azzouz

**Affiliations:** 1 Department of Gastroenterology, Mohamed Tahar Maamouri University Hospital, Faculty of Medicine of Tunis, Nabeul, Tunisia

**Keywords:** anemia, gastrointestinal bleeding, leech, Tunisia

## Abstract

Leeches are carnivorous, hermaphroditic, segmented worms mainly found in fresh water. The majority of leech attachments are external and short-lasting. Internal surfaces can be involved. Yet, esophageal attachment is very rare. We report a case of a 59-year-old female who presented with hematemesis, melena and chest pain. Upper gastrointestinal endoscopy revealed a round black foreign body in the mid-third of the esophagus identified as a leech. We carefully extracted the worm with a forceps applied to the middle of its body. The diagnosis of leech infestation should be kept in mind when exploring gastrointestinal bleeding in patients living in rural areas and those with poor living conditions.

## Plain language summary

Sanguinivorous leeches are a subgroup of annedlids that occasionally enter the human body through natural orifices and cause internal bleeding depending on the attachment site. This article presents a rare case of gastrointestinal bleeding caused by an esophageal leech infestation in a 59-year-old woman. The worm was carefully extracted with a forceps and the patient was educated about the danger of consuming or swimming in untreated water. Leech infestation should be kept in mind as a differential diagnosis when exploring gastro-intestinal bleeding or anemia in leech-endemic areas.

Leeches represent a class of segmented worms, mainly living in fresh water, with approximately 600 species [1]. Yet, it is the sanguinivorous species that cause all the fascination and repulsion of leeches. These hermaphroditic parasites attach themselves to hosts, typically externally, to feed on their blood. Internal attachment is less common and is predominantly reported in the Mediterranean region, Africa and Asia [2]. Leech infestation occurs through consumption of water from quiet streams and pools. Internal attachment was reported in the vagina and the urinary, upper respiratory and GI tract [1]. The esophageal involvement seems to be rare though. The clinical presentation of an internal leech infestation mainly depends on the exact location of the attachment. While recurrent epistaxis is the most common type of endoparasitism, gastrointestinal bleeding rarely reveals leech infestation and is exceptionally secondary to esophageal attachment. We report here a very rare case of GI hemorrhage caused by an esophageal leech that was successfully removed endoscopically.

## Case report

**Figure 1.**
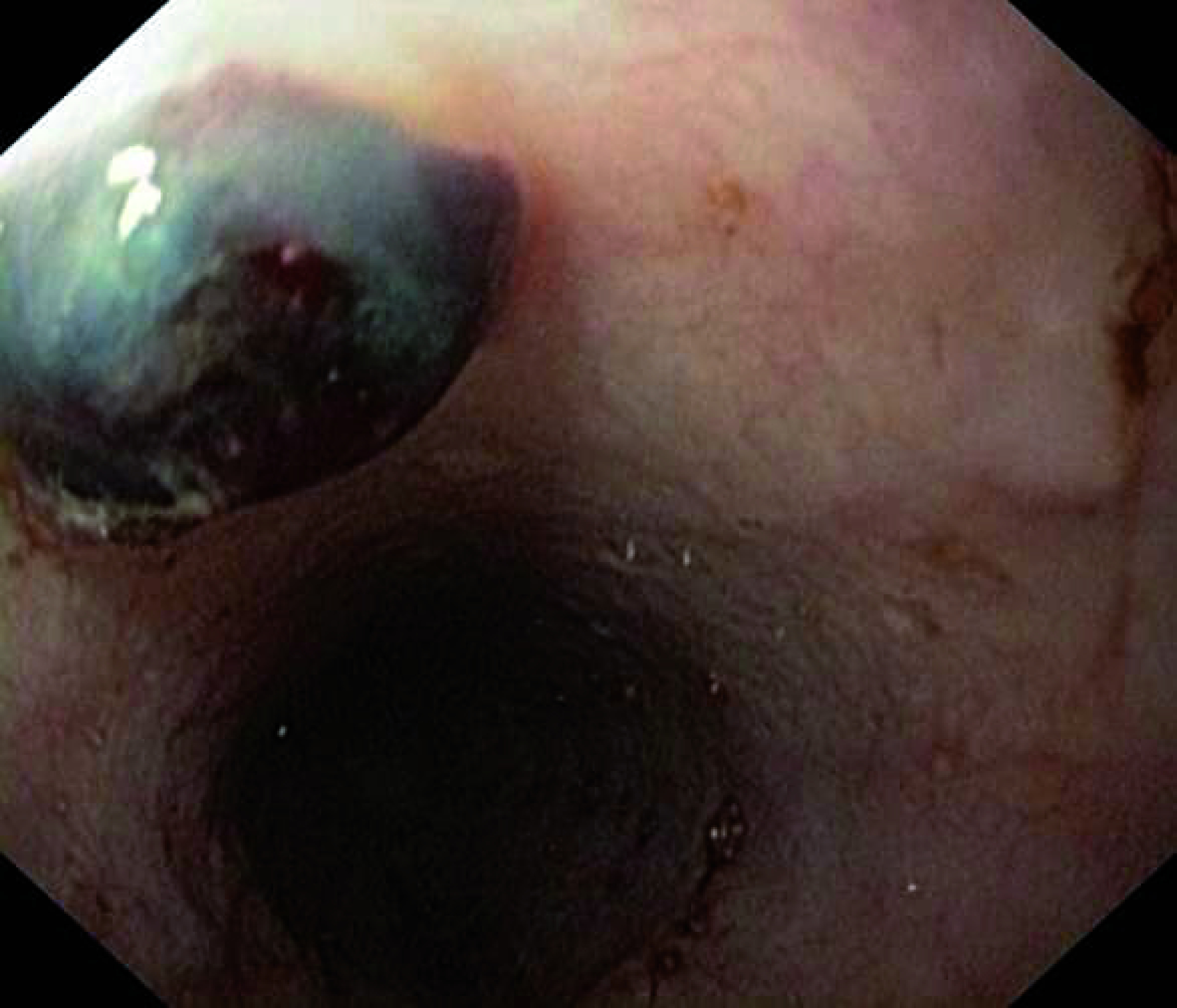
Upper gastrointestinal endoscopy in a 59-year-old woman presenting with melena and hematemesis revealing a round black foreign body in the mid-third of the esophagus identified as a leech.

**Figure 2.**
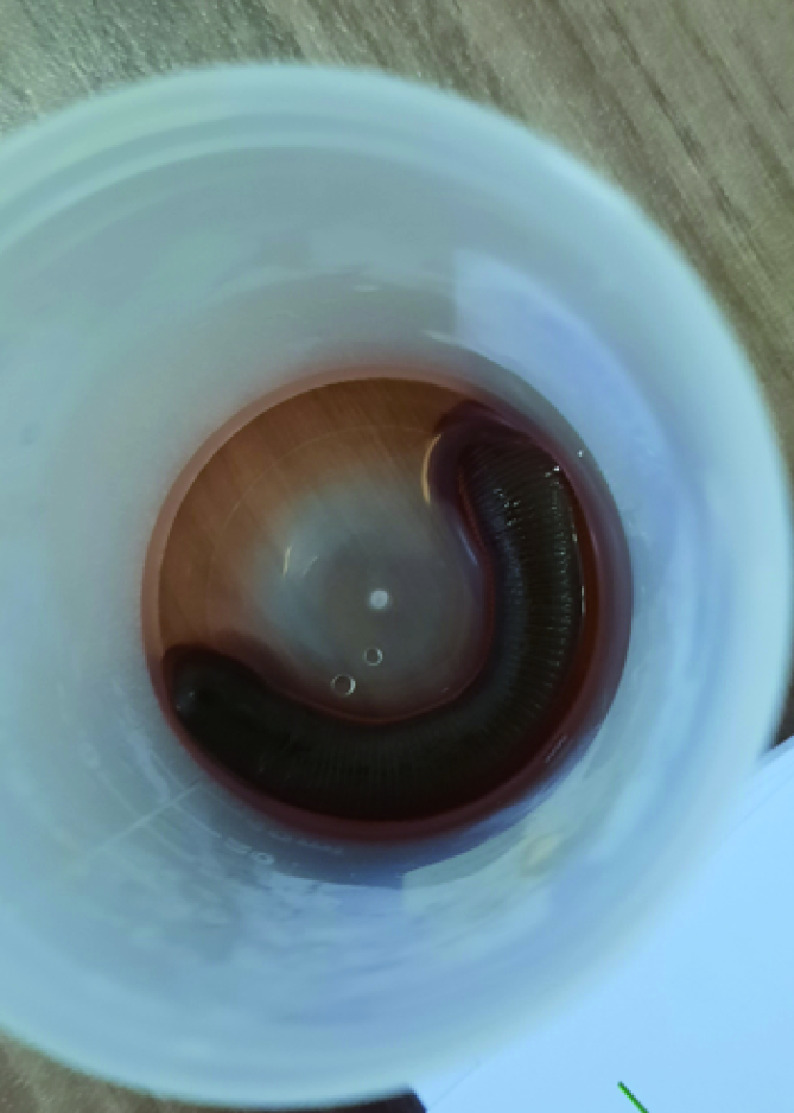
Leech (6 cm) after endoscopic removal with forceps.

A 59-year-old woman living a rural area presented to the emergency room with a 3 day history of nausea, hematemesis, melena and chest pain. The patient's medical history was unremarkable. She reported regular ingestion of water from open trough. The patient vital signs were within normal range. The rest of the clinical examination was unremarkable. Emergency electrocardiography and troponin levels were normal. The laboratory work-up revealed normocytic anemia (hemoglobin level: 7 g/dl). Upper gastrointestinal endoscopy revealed a round black foreign body in the mid-third of the esophagus with no stigma of bleeding (Figure 1). No lesions were found in the stomach and the duodenum. Careful examination of the foreign body revealed a mobile organism identified as a leech. We carefully extracted the worm with a forceps applied to the middle of its body. The leech measured 6 cm (Figure 2). The chest pain resolved immediately after the leech removal. The patient was kept under surveillance for 3 days. She did have not any bleeding recurrence. About 1 month later, she presented for a routine visit. She had remained asymptomatic and had no change in her hemoglobin level. The patient was thoroughly educated about the danger of consuming or swimming in untreated water.

## Discussion

Esophageal leeches are an exceptional cause of GI bleeding which makes this case a very rare one. Leeches are carnivorous, hermaphroditic, segmented worms. They are mainly found in fresh water but can may inhabit marine water or live on land. Leeches form a subtype of annelids characterized by the presence of front and rear suckers. The latter is used for substrate attachment, while, in bloodsucking species, the front sucker contains jaws with sharp teeth. In a single bite, leeches can ingest up to tenfold their body volume. This would normally take over a half hour but can satiate the leech for over 12 months [1]. The majority of leech attachments are external and short-lasting. Yet, internal surfaces can be involved including eyes, ears, oropharynx, the upper respiratory, urogenital and GI tracts, such is the case in our patient. In popular culture, leeches symbolize horror and fear. Yet, for a long time, these worms have been used for medicinal purposes. In fact, in ancient Chinese medicine, leeches were dried and were externally used to heal injuries and were prescribed orally for female patients to relieve several gynecological symptoms. In western medicine, leeches were used for bloodletting, a very common practice in the middle ages. This led to the creation of an occupation called the leech collectors [3]. By the end of the 19th century, leeches use had declined significantly but only to reappear again with the emergence of contemporary reconstructive surgery. In 2004, the US FDA approved leech use in plastic surgery to treat venous obstruction or muscles flaps [4]. Furthermore, leeches are currently considered to be a major natural source of anticoagulants [3]. In fact, leeches saliva contain biologically active anticoagulants, of which the main one is hirudin [5]. This factor blocks fibrin clots formation during all the feeding process by inhibiting the transformation of fibrinogen to fibrin. This would result in either internal or external bleeding in the host, depending on the attachment location, that typically lasts for 10 h but may persist for up to 7 days [5]. Blood loss is in fact the main symptom of leech infestation with a clinical presentation ranging from mild occult bleeding to significant and potentially life-threatening hemorrhage. We reviewed the literature for cases of gastrointestinal bleeding related to leech infestation. We were able to identify 12 cases [6–17]. The clinical presentation included melena, hematemesis and hematochezia. Interestingly, in nearly all cases of leeches presenting with hematemesis, the worm was located in the nasal cavity, the pharyngeal or the hypopharyngeal walls [7,10,11,16,18]. On the other hand, the esophageal site seems to be rare. To the best of our knowledge, a single case of esophageal leech infestation was published [19]. It was a 72-year-old female who, similarly to our patient, experienced retrosternal discomfort. About 3 days later, a leech extruded from her mouth and upper GI endoscopy revealed the leech former attachment site in the distal esophagus [19].

In addition to bleeding, other symptoms of internal attachment include obstruction of the involved orifice, dysphonia, dyspnea, dysuria, cough, pallor, abdominal discomfort and sensation of foreign body movement [1]. When a single external leech is diagnosed, several authors suggest further investigations for additional sites owing to the high risk of bleeding associated with leech infestation [1,20,21]. It is not clear though if a full endoscopic investigation is required in cases of external leeches or cases of single internal ones. Among cases of internal attachment and GI bleeding that we reviewed, all symptoms resolved after the leech removal and the patients remained asymptomatic with a stable hemoglobin level during the follow-up period, which was the case for our patient [10,16]. This probably indicates that internal leeches are most likely to be unique.

Management of leeches include a full history and physical examination. Internal leeches can be removed with forceps application [22]. This procedure can be challenging as leeches bodies strongly attach to mucosa, can easily rupture when pulled and are quite slippery. Endoscopists should be both patient and careful when removing leeches.

## Conclusion

Owing to the progress in sanitation and drinking water sources, internal leech infestation has become less common. Yet, it should be kept in mind as a differential diagnosis when exploring gastrointestinal bleeding in leech-endemic areas, in patients living in rural areas and those with poor living conditions. Moreover, it is crucial to establish large scale public programs empowering citizens and communities with access to sustainable safe water and sanitation services.

**Executive summary**

- Leech infestation may cause severe complications such as persistent bleeding.
- Leech infestation should be considered when exploring anemia or gastrointestinal bleeding in patients with poor living conditions or from leech-endemic areas.
- Prompt measures should be taken to educate people and provide safe and adequate water supply and sanitation in leech-endemic areas.
